# Epidemiological features of a recent zoonotic cutaneous leishmaniasis outbreak in Zagora province, southern Morocco

**DOI:** 10.1371/journal.pntd.0007321

**Published:** 2019-04-09

**Authors:** Adil El Hamouchi, Othmane Daoui, Mouad Ait Kbaich, Idris Mhaidi, Sofia El Kacem, Ikram Guizani, M’hammed Sarih, Meryem Lemrani

**Affiliations:** 1 Laboratory of Parasitology and Vector-Borne-Diseases, Institut Pasteur du Maroc, Casablanca, Morocco; 2 Molecular Genetics and Immunophysiopathology Research Team, Health and Environment Laboratory, Hassan II University of Casablanca, Aïn Chock Faculty of Sciences, Morocco; 3 Laboratory of Biology and Health, Faculty of Sciences Ben M'Sik, Hassan II University, Casablanca, Morocco; 4 Molecular Epidemiology and Experimental Pathology (MEEP)/ LR16IPT04, Institut Pasteur de Tunis, Université de Tunis El Manar, Tunisia; CSIR-Indian Institute of Chemical Biology, INDIA

## Abstract

**Background:**

*Leishmania major* is an endemic vector-borne disease in Morocco that causes zoonotic cutaneous leishmaniasis (ZCL), especially in arid pre-Saharan regions where its unique vector and reservoir are *Phlebotomus papatasi* and *Meriones shawi*, respectively, and may cause epidemics. In late 2017, the Zagora province, an endemic focus for ZCL in southern Morocco, had CL outbreak. The main objective of our investigation was to analyze the epidemiological features of this latest ZCL outbreak.

**Methodology/Principal findings:**

We analyzed epidemiological features of this latest ZCL outbreak. The Regional Delegation of Health, Zagora, recorded 4,402 CL patients between October 2017 and end of March 2018. Our findings showed that 24 municipalities were affected and majority (55.1%) of infected cases belonged to the Tinzouline rural municipality. Majority of patients were females (57.2%). While all age group patients were affected, those aged <10 years were the most affected (42.1%). During this outbreak over 5 days in December 2017, we conducted a survey in Tinzouline and recruited and sampled 114 CL patients to confirm CL diagnosis by parasitological (direct examination and culture) and molecular (ITS1-PCR) methods and identify the etiological agent of infection using ITS1-PCR-RFLP and sequencing. We completed a detailed questionnaire including clinical and epidemiological data for each patient and found 72.8% of patients presenting multiple lesions (≥2), with an average number of lesions of 5.16 ± 0.5. Lesions were more prevalent in the upper limbs, with the most common type being the ulcerocrusted lesion (60.5%). We detected no associations between lesion type and patients’ sex or age.

**Conclusions/Significance:**

Among 114 clinically diagnosed CL patients, we confirmed 90.35% and identified *L*. *major* as the species responsible for this outbreak. Self-medication using various products caused superinfection and inflammation of lesions and complicated the diagnosis and treatment. Thus, ZCL remains a major public health problem in the Zagora province, and commitment of all stakeholders is urgently required to implement a sustainable regional control program.

## Author summary

Zoonotic cutaneous leishmaniasis (ZCL) caused by *Leishmania major* is endemic in Morocco, especially in the arid pre-Saharan regions. Its main characteristics are rurality and proneness to epidemics. Zagora province an endemic focus for ZCL has been recently the scene of an important CL outbreak. Data analysis of 4,402 CL patients recorded in Zagora province, from October 2017 till Mars 2018 showed that 24 municipalities were affected to different extents; the important percent of cases belonged to Tinouzline, a rural municipality. While all age group patients were affected, children under 10 years old were the most affected. Majority of patients were female; the disfiguring CL scars often lead to the stigmatization of women and impact on their psychosocial lives. Lesions were diverse and pleiotropic ranging from a single lesion to multiple disfiguring sores, with, in extreme cases, as many as 35 lesions. *Leishmania major* was identified as the *Leishmania* species responsible of this outbreak. Self-medication by using various products caused superinfection and inflammation of lesions and complicated the diagnosis and treatment. Thus, ZCL remains a major public health problem in Zagora province, and commitment of all stakeholders is urgently required to implement a sustainable regional control program.

## Introduction

Leishmaniases, a spectrum of diseases that are caused by several species belonging to the genus *Leishmania*, are transmitted to humans and other mammals by phlebotomine sandflies. Although they cause relatively low mortality, they are responsible for considerable morbidity [1].

A dermal infection known as cutaneous leishmaniasis (CL), which is caused mostly by *Leishmania major*, *L*. *tropica*, and *L*. *infantum*, in the Middle East and North Africa, is the most common form of leishmaniasis and one of the so-called neglected diseases, which mainly affects the world's poorest populations [2,3].

The incidence of CL, endemic in 87 countries, is constantly on the rise owing to many environmental and socioeconomic factors [4,5]. According to the WHO, 90% of the recorded CL cases worldwide are from 12 countries: Afghanistan, Algeria, Brazil, Colombia, Iraq, Iran, Morocco, Peru, Sudan, Syria, Tunisia, and Yemen [6].

In Morocco, three species—*L*. *major*, *L*. *tropica*, and *L*. *infantum*—are responsible for CL. *L*. *major*, the principal etiological agent of zoonotic cutaneous leishmaniasis (ZCL), has been known to exist in Morocco since 1914. Six decades later, Rioux and Peter identified the first epidemic foci in the southeastern part of the kingdom [7]. ZCL is largely confined to arid pre-Saharan regions, where the unique vector and reservoir of *L*. *major* are *Phlebotomus papatasi* (Sandfly) and *Meriones shawi* (Shaw's Jird), respectively [7,8]. The rodent *M*. *shawi*, a *Gerbillidae* infesting the oasis-village complex in the southern Morocco was found to be parasitized by *L*. *major* since 1982 [9]. In this animal, discrete chronic lesions are almost found on the edge of the ear and rarely on the tail. The infection remains localized to the skin for several months or even years; it is often necessary to wait for the pre-mortem stage to see cutaneous or visceral metastases [7]. Moreover, in the arid areas, the burrows of these rodents provide microhabitats suitable for sand flies breeding and larvae development [9,10]. A closely related association between the eruption of *M*. *shawi* population and the incidence of ZCL was reported in endemic areas in Morocco [11]. Typically, the foci of ZCL are either palm groves or periurban areas with unhygienic habitats [12].

Clinical manifestations of ZCL are particularly diverse and pleotropic, ranging from a single self-limiting lesion to multiple disfiguring lesions, with, in extreme cases, as many as 30 lesions [13]. The establishment of a primary *Leishmania* infection and the development of a dermal disease are currently believed to depend on the parasite’s genetic background, host immune response, and factors related to the sandfly. All these components interact in a close relationship to produce different clinical forms [14,15].

According to the available records from the Moroccan Ministry of Health, between 2000 and 2016, a total of 31,354 cases of ZCL were reported [16]. In Morocco, ZCL appears to shift between alternating endemic and epidemic cycles, with the latter involving brutal outbreaks lasting two or three years and the former involving long remission periods of about five years or more, during which most affected people are children and newcomers [16,17]. Following outbreaks, however, the deceleration of control measures creates a higher risk of severe epidemics in endemic areas.

The province of Zagora, a pre-Saharan region in southern Morocco, is an endemic focus for ZLC, where the last outbreak between 2008 and 2010 included 4,437 cases [18]. Late in 2017, reports emerged on a new outbreak with a high incidence in the region. In our study, we aim to understand the epidemiological characteristics of this latest outbreak and to shed light on the features of the ZCL outbreak in order to enable more focused and engaged control procedures to prevent new outbreaks in the future.

## Materials and methods

### Study area

**Fig 1:**
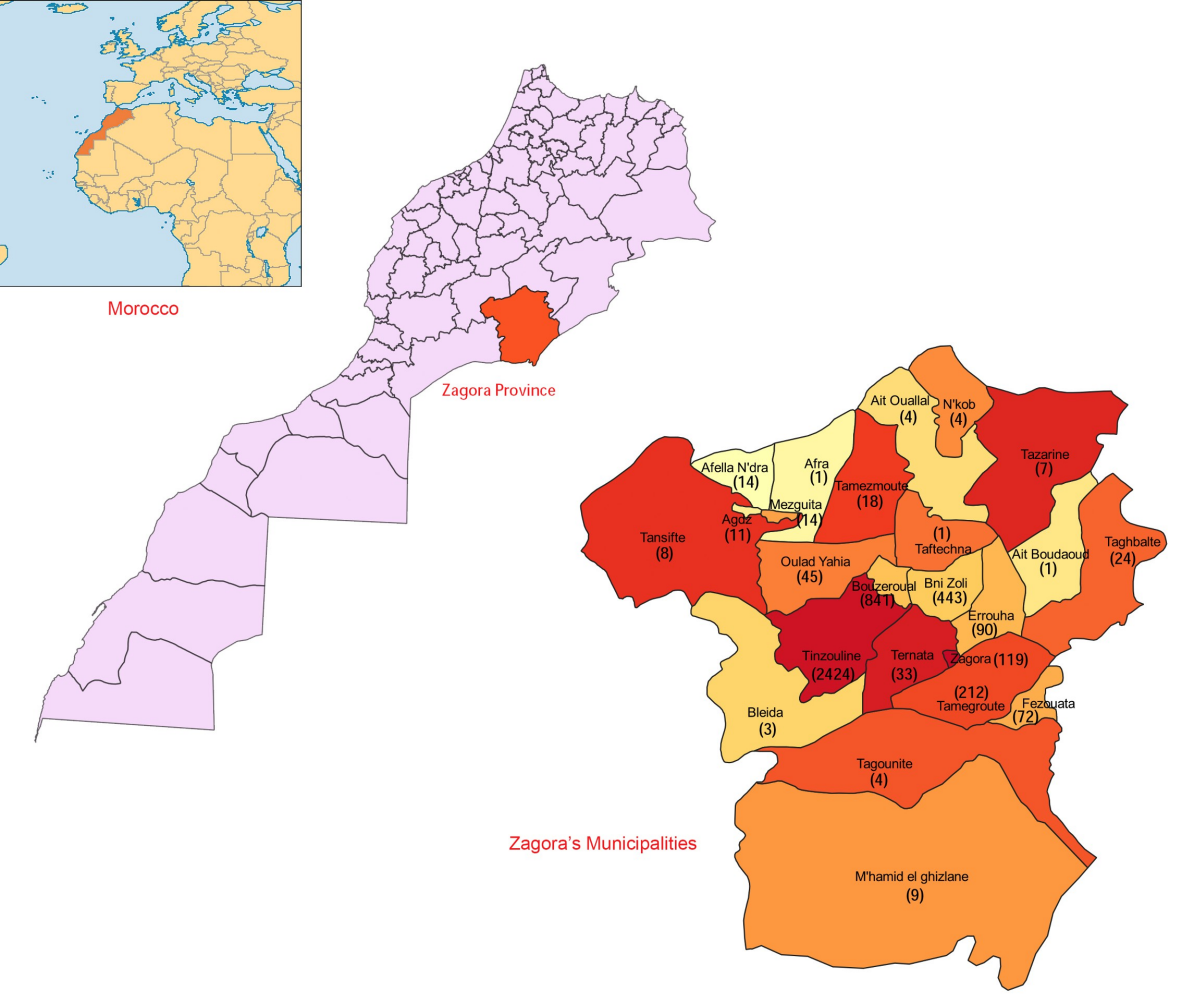
Geographic locations of municipalities within the province of Zagora (Morocco) affected by zoonotic cutaneous leishmaniasis. **The numbers between brackets indicate the ZCL cases recorded in the municipalities**. The maps were generated by ourselves using the open source software QGIS 3.2 (https://www.qgis.org). The shapefiles map layers of the administrative subdivision of Morocco are kindly provided for free download by the site web: https://www.saidgis.com.

We conducted this study in the province of Zagora, in the region of Drâa-Tafilalet in southern Morocco (Fig 1), a territory dominated by the chain of Anti-Atlas Mountains. The climatic environment is generally part of the Saharan bioclimatic stage, with very low average annual rainfall, decreasing from the north to the south, from 100mm at Agdz to 60 mm at Zagora. The rainy periods fall between September and May, with 30–40 rainy days annually.

Tinzouline, a rural municipality in the province of Zagora, is situated at an altitude of 1,051m at around 30°30′26.1″N 6°06′07.9″W, with a total population of about 15,000 inhabitants. This municipality is characterized by severe winters (temperature ranging from −1°C to −7°C) and hot summers (40°C). Located 35km away from Zagora, this village was the scene of a ZCL outbreak in 2017; the first cases began to appear from October 2017. Besides Tinzouline, 23 other municipalities were affected to different extents by ZCL infections (Fig 1).

### Statistical analysis of patients’ data during the ZCL outbreak

Between October 2017 and March 2018, the regional health authority of Zagora province recorded 4,402 patients with ZCL; from these records, we extracted the age, sex, and address of each patient. From the onset of the outbreak, the CL cases were diagnosed by parasitological method (direct microscopic examination). Thereafter, giving the large number of infected people, the passive and active case-detection of CL was based only on clinical features.

We carried out descriptive statistics, a Chi-square (χ2) test, and Fisher’s exact test using Prism 7 software (GraphPad Software, Inc., La Jolla, CA, USA) and SPSS v.20 (SPSS, Inc., Chicago, IL, USA). Statistical significance was defined at P < 0.05. The 95% confidence intervals (CI) for the positivity rates were calculated using the Wilson method [19], implemented in the online program EpiTools epidemiological calculators [20].

### Patients’ recruitment and sampling

During a period of five days in December 2017, we recruited 114 patients at the health center of Tinzouline. All recruited patients presented skin lesions clinically suggestive of CL, and never treated by Glucantime injection. Pregnancy women, patients presenting chronic illness (eg, blood pressure issues, diabetes, etc.) were not skin-sampled.

We interviewed each patient using a structured questionnaire comprising all the information about the patient (patient’s code, age, gender, address, and travel history) and the disease (the onset of the lesion, diagnosis, history of treatment, and number and location of lesions). We consolidated all completed questionnaires for data input and analysis.

We performed patient sampling for *Leishmania* cultures and smear staining by dermal scraping of the lesion’s edge. For molecular CL diagnosis and identification of *Leishmania* species, we used the swab sampling method, which is painless and simple to perform. We took swab samples by gently rubbing over the skin lesion approximately five times and then storing them at −20°C until DNA extraction.

We fixed and stained all lesion smears with absolute methanol and Giemsa (Avicenne Group, Casablanca, Morocco), respectively, for CL direct diagnosis through microscopic examination. We analyzed all slides twice using a 100x immersion objective.

We cultured *Leishmania* on an RPMI 1640 medium (Biowest, Nuaillé, France) supplemented with 2mM L-glutamine (Eurobio, Les Ulis, France), 10% fetal bovine serum (Biowest, Nuaillé, France), and 1% penicillin/streptomycin (100U/mL penicillin and 100μg/mL streptomycin; Biowest, Nuaillé, France), followed by incubation at 25°C.

### DNA extraction

We performed DNA extraction from a cotton swab. We placed each swab in a 1.5mL centrifuge tube containing 250μL of a lysis buffer (50mM NaCl, 50mM Tris, and 10mM EDTA; pH = 7.4), 1% SDS, and 100 μg/mL proteinase K. After incubating the lysates overnight at 60°C, we subjected them to phenol–chloroform extraction, followed by ethanol precipitation, as described elsewhere [10].

We then quantitatively determined the DNA samples using NanoDrop (Thermo Fisher Scientific, Waltham, MA, USA) before dilution to a final concentration of 50ng/μL.

### Detection and identification of *Leishmania* species by ITS1-PCR-RFLP

We used LITSR and L5.8S primers to amplify ITS1 according to the protocol described by Schonian et al. in 2003 [15], using a negative control (without DNA) for each PCR run. In order to identify *Leishmania* species, we subjected the positive PCR products of 350bp to enzymatic restriction by *HaeIII* (New England Biolabs, Hitchin, UK) for 2h at 37°C. We analyzed RFLPs using electrophoresis on a 3% agarose gel containing ethidium bromide, using a 100bp DNA size marker (HyperLadder 100bp Plus; Bioline, London, United Kingdom). We compared the restriction profiles to the profiles of Moroccan strains previously identified by sequencing as *L*. *infantum*, *L*. *major*, and *L*. *tropica*.

### DNA sequencing and phylogenetic analysis

We directly sequenced 10 randomly chosen ITS1-PCR products to confirm our PCR-RFLP identification results. We purified them using the Exonuclease I/Shrimp Alkaline Phosphatase (GE Healthcare, Chicago, IL, USA) and then sequenced them using BigDye Terminator v3.1 Cycle Sequencing Kit (Applied Biosystems, Foster City, CA, USA) and an ABI Prism 3130 DNA automated sequencer (Applied Biosystems).

Phylogenetic analysis of 10 *L*. *tropica* ITS1 sequences, generated in this study, was performed to confirm the identification of *L*. *major* as etiological agent, responsible of the outbreak occurred in Zagora province. In addition to our ten ITS1 sequences, other ITS1 *L*. *major* sequences and additional *Leishmania* spp. retrieved from GenBank database were used. For data analysis we used the MEGA version 7 (http://www.megasoftware.net); phylogram was constructed using the Maximum likelihood algorithm with the Jukes-Cantor model. The tree topology was supported by 1000 bootstrap replicates.

### Ethics statement

Written informed consent was obtained from all the adults who participated in the study. Consent for inclusion of young children, was obtained from parents or guardians. The study and the protocols were approved by the Ethics Committee for Biomedical Research (CERB) of the Faculty of Medicine and Pharmacy, Rabat, Morocco.

## Results

### Patient data analysis

Between October 2017 and the end of March 2018, the Regional Delegation of Health, Zagora, clinically diagnosed and recorded a total of 4,402 patients with skin lesions from Zagora as cases of ZCL. All patients were freely treated with Glucantime (meglumine antimoniate) according to the Moroccan Ministry of Health guidelines.

24 municipalities were affected to different extents; the most affected area during this CL outbreak was the rural municipality of Tinzouline, with 55.1% (n = 2424) of the total CL cases, followed by Bouzeroual and Bni Zoli, with 19% (n = 841) and 10% (n = 443), respectively; the 21 other municipalities recorded a range of 5% (n = 212) to 0.02% (n = 1) of the total cases reported (Fig 1).

Almost all ZCL cases were from rural municipalities, with only 2.7% from the urban city of Zagora (Fig 1). Among the 4,402 patients with ZCL, 2,517 cases were females (57.2%) and 1,885 were males (42.8%) (Table 1), a statistically significant gender difference (χ^2^ = 90.73, *P*<0.0001).

**Table 1:** Distribution of patients with zoonotic cutaneous leishmaniasis by the age group and sex.

Age group (years)	Sex		Total
	Male	Female	
0–10	959 (21.8%)	895(20.3%)	1854 (42.1%)
11–20	560 (12.7%)	588 (13.4%)	1148 (26.1%)
21–30	134 (3.0%)	408 (9.3%)	542 (12.3%)
31–40	121 (2.7%)	304 (6.9%)	425 (9.7%)
41–99	111 (2.5%)	322 (7.3%)	433 (9.8%)
Total	1885 (42.8%)	2517 (57.2%)	4402 (100.0%)
*P*-value	<0.0001		

Patients with ZCL were aged between two months and 99 years, with an age median of 13 years (interquartile range: 7–27 years), and the majority of the patients were under 10 years old (Table 1).

### Clinical, parasitological, and molecular analysis of patients with ZCL (*n* = 114)

#### Clinical data of patients with ZCL

During this outbreak, for a period of five days in December 2017, we conducted a survey in Tinzouline, the municipality where the number of ZCL cases was very high. We recruited a total of 114 patients with CL (76 females, 38 males). The clinical characteristics of patients with CL are summarized in Table 2.

**Table 2:** Clinical characteristics of sampled patients with zoonotic cutaneous leishmaniasis (*n* = 114).

	Number of patients	%
**Type of lesion**		
Ulcerocrusted	69	60.5
Papulonodular	31	27.2
Ulcer	14	12.3
**Number of lesions**		
1	31	27.2
2–5	49	43.0
6–9	18	15.8
≥10	16	14.0
Distribution of lesions*		
Face	61	53.50
Upper limbs	78	68.42
Lower limbs	39	34.21
Trunk	6	05.26

*The sum of the distribution of the lesions is not equal to the total number owing to the presence of more than one lesion in some patients.

72.8% of patients with CL presented multiple lesions (two lesions or more), whereas 27.2% presented a single lesion. The average number of lesions per patient was 5.16 ± 0.5 (range: 1–35). Regardless of the number of lesions, the upper limbs were the most affected area, with 68.42% of the patients having lesions on their upper limbs, whereas the face was the second most common area, with 53.50% of the patients having CL lesions on their faces (Table 2). The difference between these two areas of infection was statistically significant (χ^2^ = 4.115, *P* = 0.042).

During this ZCL outbreak, we encountered a number of different varieties of lesions and complex forms. In addition to the very large number and size of lesions that we found in one person, the types of lesions were heterogenic, but the most common lesion was the ulcerocrusted form found in 60.5% of patients with CL. The two other forms that we found were papulonodular (27.2%) and ulcer (12.3%) forms (Table 2).

**Fig 2:**
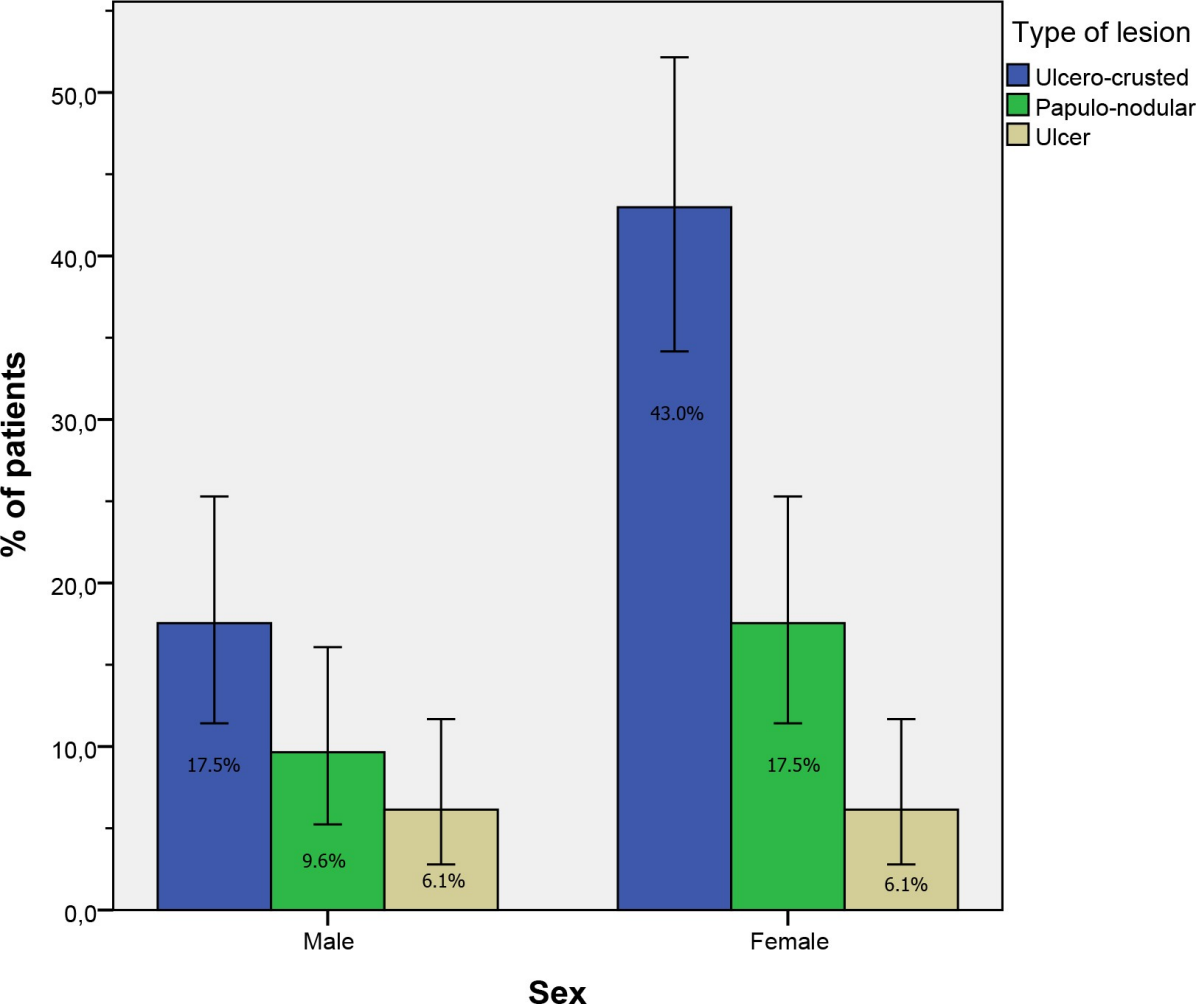
Type of lesion and sex distribution of patients with zoonotic cutaneous leishmaniasis (*n* = 114). The error bars represent 95% confidence intervals.

**Fig 3:**
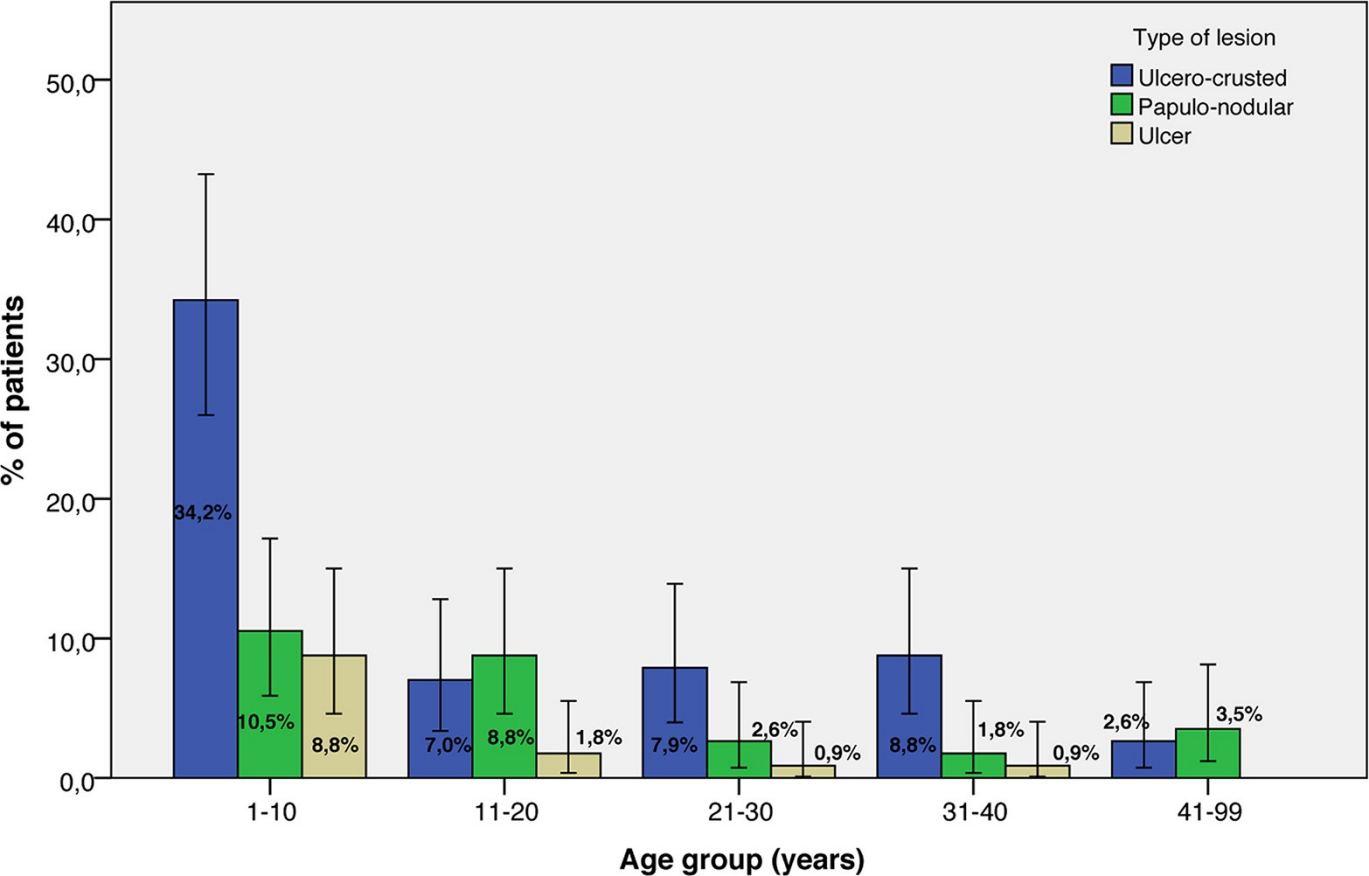
Type of lesion and age group distribution of patients with zoonotic cutaneous leishmaniasis (*n* = 114). The error bars represent 95% confidence intervals.

The distribution of lesion types by sex and age group is presented in **Figs** 2 and 3, respectively. We detected no associations between the type of the lesion and sex (*P* = 0.337, Pearson’s chi-square) or the age group of the patients (*P* = 0.151, Fischer’s exact test).

#### Parasitological and molecular diagnostic results

We used three diagnostic methods to confirm the clinical diagnosis of the 114 sampled patients with CL: direct examination, culture, and ITS1 amplification by PCR. We performed slide smears and *Leishmania* isolation cultures using dermal scraping products, whereas for molecular analysis, we used swabs. We did not apply the three methods in some patients for *Leishmania* diagnosis because of their vulnerability owing to their young age, inflammatory or superinfected lesions, or the presence of other diseases (e.g., diabetes, blood pressure).

We confirmed CL in 103 out of the 114 patients (Overall positivity rate: 90.35%) by reference gold standard diagnosis (microscopic detection of amastigotes in Giemsa-stained smears and/or culture isolation of *Leishmania*) and/or ITS1-PCR. The positivity rate of each method of diagnosis used is presented in Table 3. We found the positivity rate by microscopic detection of amastigotes on stained smears and ITS1-PCR amplification to be almost equal (≈72%). The cultures of scraped skin tissues from 91 patients had a positivity rate of 59%. Test of significance (chi-square test) showed that there was no significant difference between the three diagnostic methods (χ^2^ = 4.384, *P* = 0.111). The detailed results obtained are presented in S1 Table.

**Table 3:** Positivity rates of the three diagnostic methods used for the 114 sampled patients.

	Positive	Negative	Total	Positivity rate (%)	95% CI
Microscopy	71	29	100	71.00	61.46–78.99
ITS1-PCR	80	31	111	72.07	63.10–79.57
*In vitro* culture	54	37	91	59.34	49.07–68.86
Parasitological techniques* and/or PCR	103	11	114	90.35	83.54–94.53

CI: Confidence interval

* Parasitological techniques: Microscopy and/or *in vitro* culture

#### Molecular identification of *Leishmania* species by ITS1-PCR-RFLP

Species identification by *Hae*III-RFLP digestion of all ITS1-PCR products allowed us to identify *L*. *major* as the species responsible for the outbreak that occurred in Zagora province. By sequencing 10 ITS1-PCR products, we also confirmed the identity of *L*. *major*. The ITS1 sequences (339 bp) were deposited in GenBank database [accession numbers: MH932563-MH932572].

**Fig 4:**
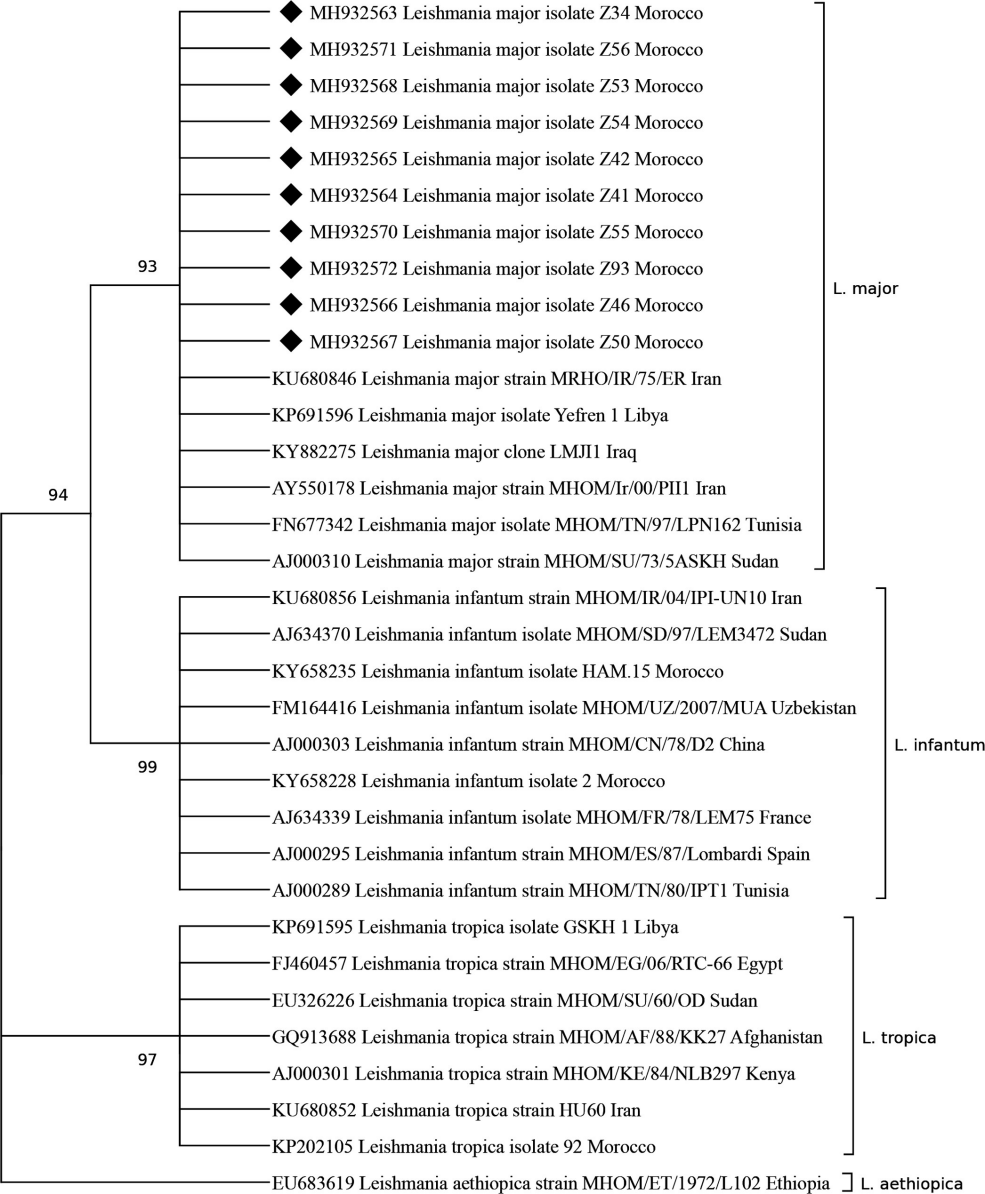
A Maximum Likelihood consensus tree comparing 339 bp ITS1 *L*. *major* fragments from Zagora province [MH932563-MH932572] with other *L*. *major* strains and Old World *Leishmania* spp. The number of bootstrap replicates are 1000 and branches corresponding to less than 80% bootstrap replicates are collapsed. The percentage of replicate trees in which the associated taxa clustered together in the bootstrap test (1000 replicates) are shown next to the branches. The GenBank accession numbers, *Leishmania* sp. and country of origin are included for each sequence. Black diamonds indicate ITS-1 sequences of *L*. *major* strains isolated from Zagora province. The other ITS-1 sequences were extracted from the GenBank database.

The phylogenetic analysis based on the 10 ITS1 sequences generated in this work and other sequences of *Leishmania* spp. available in Genbank database, revealed that the Zagora’s *L*. *major* sequences clustered together with other *L*. *major* strains from different African and Asian countries, separately from *L*. *tropica* and *L*. *infantum* which appear in two different and well-supported clusters (Fig 4).

## Discussion

Since leishmaniasis became a notifiable disease in Morocco in 1996, the number of cases in the province of Zagora has been counted by the hundreds. Between 2002 and 2003, a lull of two years was noted, with 22 and 52 cases, respectively [16]. The next outbreak was triggered in 2008 with 1,421 cases, increasing to 1,882 cases in 2009 and 1,134 cases in 2010. A very significant steady drop in the number of leishmaniasis cases to 48 cases in 2015 followed this epidemic, before the latter outbreak reported in this work erupted during the last quarter of 2017. The abruptness of these epidemiological waves and the length of the interepidemic silences cannot be explained without referring to the periarid rainfall regime. This climatic type is characterized by long periods of drought, interspersed intermittently with violent storms that mark the biological cycles profoundly. With the rain, vegetation is the first to react, showing a strong push, followed by the proliferation of rodents and vectors [7,21], after which *L*. *major*, hitherto sporadic, multiplies actively. The epizootic precedes the epidemic [7]. Indeed, the inhabitants of Tinzouline, the most affected municipality, reported that the density of the *M*. *shawi* rodent reservoir population had become very high; the sylvatic cycle occurring at poor dwellings in different areas within the municipality. Deceleration of the control measures implemented in this province, following the decrease in ZCL cases in the years preceding this outbreak, could also be a risk factor responsible for this outbreak (e.g., dump pits, open sewerage, and cattle manure in the vicinity of dwellings [microfocus]).

Apart from causes, such as environmental conditions, socioeconomic status, and human behaviors, the increase in human leishmaniasis prevalence is mainly attributed to several demographic risk factors, commonly including sex, age, household design, and construction material [2,3].

The affected age range is reported to depend on the intensity of transmission (force of infection) to which populations are exposed [22]. Our data revealed that all age groups were affected. However, children under 10 years old displayed the highest rate of infection (42%), while groups above 31 years old showed the lowest rate of infection (9.7%). In established endemic areas, the prevalence of CL was reported to increase generally with age up to 15 years, after which it stabilized, probably reflecting the progressive buildup of immune protective status [2].

Our analysis showed that both sexes were affected; however, women were more affected than men, with the most important difference according to gender observed in groups aged 20 years and above. This factor is related to behavioral patterns that increase the exposure of people to the vector, in our case *P*. *papatasi*, the proven vector of *L*. *major* in North Africa and the Middle East [23]. During the hot summer nights characterizing this pre-Saharan region, men are known to stay and sleep outdoors (i.e., on terraces), unlike women, who are often indoors. As *P*. *papatasi* is highly endophilic and anthropophagic [24], women are more susceptible to being bitten by the vector, which may explain the predominance of females infected by ZCL in this study. Regardless of the reasons behind this fact, ZCL may cause cosmetic disfigurement and permanently disfiguring scars, which may create lifelong stigma and impact women’s lives [25,26]. In studies carried out in Moroccan rural ZCL foci, substantial gender differences related to the perceived burden and psychosocial consequences of ZCL were reported, in which women seemed to be more strongly stigmatized and the emotional well-being of young single women with facial lesions was strongly affected by ZCL scars [27,28]. The emotional representations associated with ZCL among women were also demonstrated to be correlated with the loss of self-esteem and feelings of inferiority [29].

Our clinical analysis of the 114 patients with ZCL showed that the ulcerocrusted lesion form was the most frequent compared to the papulonodular and ulcer forms; the size of the lesion was also very variable. However, what caught our attention was the striking number of lesions observed, in some cases reaching up to 35 lesions. The lesions frequently appeared severely inflamed and superinfected because most patients attempted to treat themselves using different kinds of spices (salt, chilies, etc.), herbs (henna, white wormwood), sand, soil, or toxic products (tar, bleach water, tobacco, and used engine oil), leading to superinfection of lesions and complication of the biological diagnosis and treatment.

Clinical manifestations of *Leishmania* infections depend on multifactorial parameters, such as human genetic susceptibility and the genetic background of the parasite. The factors related to the vector may also affect the CL manifestations [30]. By taking multiple blood meals and multiple inoculations, the vector increases its capacity to transmit parasites, resulting in multiple lesions on the susceptible host, which can lead to disfiguring scars; these forms are often difficult to treat and require specialized advice [31]. Lesions were more common in the exposed parts of the body (face, upper and lower limbs), which appear to be more prone to sandfly bites. However, the location of predilection for *L*. *major* infection was the upper limbs. Unlike CL due to *L*. *tropica*, where most of the lesions are on the face [32,33], cutaneous leishmaniasis due to *L*. *major* is more common in the extremities [34–36].

In endemic ZCL foci, despite the decrease in the number of CL cases, the vector and rodent control measures should be vigorously maintained. In addition to control measures, awareness campaigns for a better knowledge of the disease should be conducted regularly in order to avoid exposure to infections, as well as self-medication, which is responsible for most cases of superinfection and both clinical and therapeutic complications.

Finally, operational research and collaboration among researchers, clinicians, veterinarians, and public health authorities is required to establish a suitable strategy for the control of ZCL and to prevent future outbreaks.

## Supporting information

S1 Table Detailed results of the three diagnostic methods used for the 114 sampled patients. (XLSX)
